# Special Issue “Salmonella: Pathogenesis and Host Restriction”

**DOI:** 10.3390/microorganisms9020325

**Published:** 2021-02-05

**Authors:** France Daigle

**Affiliations:** 1 Département de Microbiologie, Infectiologie et Immunologie, Université de Montréal, Montréal, QC H3T 1J4, Canada; france.daigle@umontreal.ca; 2 Swine and Poultry Infectious Diseases Research Center (CRIPA-FRQNT), Montréal, QC H3C 3J7, Canada

Bacteria of the *Salmonella* genus include several serovars that are closely related, although they can colonize different ecological niches, different hosts, and cause different diseases. Some serovars have a broad host spectrum, including mammals, insects, birds, and plants, while others are specific to only one host species. This Special Issue contains a research article on genomic comparative analyses that focus on serovar adaptation to pigs [1] and a review on the role of the iron-acquisition systems of serovars found in chickens [2], among others. 

*Salmonella* serovars either persist in an asymptomatic carrier state or cause symptomatic infection. In humans, each year, the global burden of *Salmonella* infections causing gastroenteritis is 94 million cases and approximately 155,000 deaths are caused by non-typhoidal *Salmonella* (NTS) (predominantly by serovars *S.* Typhimurium and *S.* Enteritidis) [3]. In developed countries, NTS outbreaks are often associated with contaminated processed food and fresh produce. Bacteremia arises when invasive NTS (iNTS) infections occur in individuals with immunosuppression, individuals suffering from malnutrition, or other severe infections such as malaria or human immunodeficiency virus (HIV), as well as elderly individuals and young children. High global morbidity and mortality in iNTS infections occurs in developing countries with a global burden of 3.4 million cases and over 680,000 deaths [4]. Typhoidal *Salmonella* (TS) are responsible for enteric fever, a systemic life-threatening disease, with an estimated global annual burden of between 11.9 and 26.9 million cases, resulting in 128,000 to 216,500 deaths per year [5], which include serovar Typhi causing typhoid fever, and serovars Paratyphi A, B, and C causing paratyphoid fever.

After ingestion of contaminated food or water, *Salmonella* colonizes the intestine and induces inflammation, which results in gastroenteritis. *Salmonella* invades the intestinal epithelium using a type three secretion system located on *Salmonella* pathogenicity island 1 (SPI-1) [6] and invasin proteins, Rck and PagN [7]. In this Special Issue, Lerminiaux et al. [8] have applied phylogenetic comparisons to study the distribution, evolution, and stability of SPI-1, while Wu et al. [9] have compared PagN sequences and have identified a residue that contributes to adhesion and invasion. Following invasion, *Salmonella* will spread via the bloodstream to the spleen, liver, and gallbladder, where they replicate in macrophages. Verma et al. [10] have reviewed the use of organoids as a complex model to study *Salmonella* host cell interactions. Each step of the infection process needs to be tightly regulated. The specific regulatory proteins of a two-component system will transmit the signal by phosphorelay with various output functionalities including the gene expression level of several genes to adapt to a particular environmental change. Murret-Labarthe et al. [11] investigate the role of all two-component systems in serovar Typhi.

*Salmonella* forms biofilms to persist in the environment or to resist antimicrobial peptides and antibiotics, reduce phagocytosis, and resist the innate immune system. Biofilms are defined as a highly organized multicellular community of bacteria embedded in a self-induced extracellular matrix. Here, Hahn and Gunn [12] describe the role of extracellular polymeric substances in biofilms and in host innate immunity. Furthermore, Sokaribo et al. [13] describe the expression of CsgD, the major biofilm regulator, under several conditions. The information gained in these studies will help to develop strategies to fight against *Salmonella*. 

As with many bacterial pathogens, antimicrobial resistance is also rising in *Salmonella*. Multidrug resistance has been detected in NTS isolates from animals and humans [14] including iNTS [15] and typhoid isolates [16]. Transfer of plasmids between isolates represents a common mechanism of antibiotic resistance. Emond-Rheault et al. [17] describe a procedure to detect plasmids and antimicrobial genes by whole genome sequencing.

Current advances in sequencing and bioinformatics, and the development of new models to study host cell interactions have provided important insights to determine the roles of genes and their regulation that may provide keys to combat *Salmonella* infections. 
